# Transactions of the Pathological Society of Philadelphia

**Published:** 1839-12-21

**Authors:** 


MEDICAL EXAMINER.

DEVOTED TO MEDICINE, SURGERY, AND THE COLLATERAL SCIENCES.

No. 51.] PHILADELPHIA, SATURDAY, DECEMBER 21, 1839. [Vol. II.

### TRANSACTIONS OF THE PATHOLOGICAL SOCIETY OF PHILADELPHIA.

*December 16th*, 1839.

The President, Dr. Gerhard, in the Chair.

Dr. Kerr presented a specimen of *Pericarditis* and *Endocarditis*, taken from a child nineteen months old, and read the following account of the case:

*Case of Pericarditis and Endocarditis in a Child nineteen months old—Cure of the disease by adhesions—Death by lobular pneumonia.*

The child was ill with the pneumonia for one week, but no notes were preserved. The prominent symptoms were more directly connected with the brain than with the thorax. At least, at the beginning of the disease there were convulsions of the hands and feet, with stupor. The child had been feeble for some time previously.

*Autopsy forty hours after death.*—Exterior, slight emaciation; slight rigidity of limbs; abdomen distended, tympanitic; chest anteriorly, dull upon percussion over the lower two-thirds of the left side; on the right, resonant in the upper half, dull below; many white cicatrices seen over the body, resembling those of variola.

*Brain.*—Blood-vessels of dura mater congested; externally, the membrane is covered with numerous drops of blood; arachnoid and pia mater of both hemispheres congested, especially at the superior part of the middle lobe, where, in a space of three inches square, the larger vessels are greatly distended, the minute capillary vessels injected, of a deep scarlet hue, and freely inosculate with each other. The pia mater can be raised, however, without tearing up the subjacent cortical substance, which, at this point, is of a bright violet. The cortical substance throughout is of a deeper colour than usual,—somewhat softened on the inferior portions of the anterior lobes, elsewhere of normal consistence. Plexus choroides much injected; no fluid in the left ventricle; right contains half an ounce; tenia hippocampi pulpy; milk white colour. At the base of the brain the arachnoid not perceptibly thickened; meninges less injected than at the summit; no granulations observed in the fissure of Sylvius; cerebellum normal.

*Thorax.—Right chest.* Lungs adherent to the ribs inferiorly by membranous bands; pleura highly injected; two ounces of reddish serum effused in the cavity; upper lobe normal, except along the upper margin, which is rounder than usual, from enlargement of vesicles; lower lobe, and part of the middle, congested, and exhibiting, when cut into, a marbled appearance from congestion and hepatization of several lobules separated by portions of unchanged lung.

*Left chest.*—Firm membranous adhesions between the pleura costalis and pulmonalis; four ounces of bloody serum in the chest; lobules of the lower lobe generally hepatized, friable, and sink in water; interspersed through the hepatized masses are portions of healthy lung; upper lobe emphysematous in superior portion; congested in its lower half.

*Heart.*—Tissue very firm; pericardium contains a small quantity (say half an ounce) of bloody serum. It is connected with the heart by firm membranous attachments. Anterior bands are short, opaque, and devoid of capillary vessels, whilst those which are posterior are longer, and of a deep scarlet hue, derived from numerous red capillaries. Pericardium, generally, thickened; that portion of it covering the heart is of a dull white colour; numerous straw-coloured patches are seen at different points, more especially on the auricles, where they are raised from a half to two lines above the general surface. The left ventricle being opened, a straw-coloured coagulum is found, mixed with red blood; this coagulum is very firm and elastic,—its elasticity is similar to that of thin India-rubber. The endocardium of this ventricle is thickened and opaque; in the upper portion of the ventricle, near the aortic valves, are numerous minute spots subjacent to the serous coat, of a light yellow, resembling putty in consistence and colour. The mitral valves thickened, much injected, and contain several depositions between their laminæ, similar to those just mentioned.

*Left ventricle* is partially distended with a coagulum of the same character as that in the ventricle. Thickness of the *left ventricle* at its middle, exclusive of the columnæ carneæ, four lines.

*Right ventricle*, exclusive of columnæ carneæ, one line, measured in the middle.

The lining membrane of the right ventricle and auricle of a dull white colour, with slight opaque deposits beneath the serous tissue.

*Valves.*—Semilunar opaque, bright scarlet on their edges; the same yellowish white spots are seen scattered over them.

Semilunar valves perfectly elastic, thickened in points.

Tricuspid valves close perfectly; generally healthy, with the exception of thickening on their edges.

Valves of pulmonary artery perfect, without any abnormal deposit. Valves of pulmonary artery seventeen lines in circumference; those of aorta seventeen lines.

*Abdomen.*—No effusion into the cavity of the abdomen. Peritoneum normal. Liver enlarged, six inches long, three wide, two thick, much congested; colour and consistence normal.

*Gall bladder* distended by thick, yellowish bile.

*Stomach.*—Mucous coat pale ash-colour, softened, impossible to raise strips by traction.

*Small intestines* normal.

*Kidneys.*—Right, cortical substance congested with blood; left, normal.

*Mesenteric glands* vary in size from that of a pea to a hazel-nut; bright pink internally.

*Spleen* firm, normal, two and a half inches long, one wide, one-half thick.

*Remarks.*—This case is interesting from several circumstances. From the character of the anatomical lesions, it is evidently lobular pneumonia. Pleuritis, pericarditis, and endocarditis, were the primitive affections: subsequently the brain became involved, as is evidenced by the paralysis and rigidity of the limbs. The cerebral affection at this time most probably arose from congestion of the capillaries of the meninges, since slight topical depletion by leeches restored partially the muscular power. The chain of morbid results above mentioned is not unfrequently seen in more advanced age, but it is rare to find pericarditis and endocarditis so well marked in an infant.

